# Evaluation of Wound Healing and Antibacterial Potential of *Greyia radlkoferi* Szyszyl. Ethanolic Leaf Extract

**DOI:** 10.3389/fphar.2022.806285

**Published:** 2022-04-11

**Authors:** Samantha Rae Loggenberg, Danielle Twilley, Marco Nuno De Canha, Debra Meyer, Ephraim Cebisa Mabena, Namrita Lall

**Affiliations:** ^1^ Department of Plant and Soil Sciences, University of Pretoria, Pretoria, South Africa; ^2^ Department of Biochemistry, Faculty of Science, University of Johannesburg, Johannesburg, South Africa; ^3^ Mothong African Heritage, Pretoria, South Africa; ^4^ School of Natural Resources, University of Missouri, Columbia, MO, United States; ^5^ College of Pharmacy, JSS Academy of Higher Education and Research, Mysuru, India; ^6^ Bio-Tech R and D Institute, University of the West Indies, Kingston, Jamaica

**Keywords:** *Greyia radlkoferi* szyszyl, antibacterial, *Staphylococcus aureus*, *Pseudomonas aeruginosa*, nitric oxide, human keratinocytes, wound closure, platelet derived growth factor AA

## Abstract

Angiogenesis is an essential mechanism in both physiological and pathological functions, such as wound healing and cancer metastasis. Several growth factors mediate angiogenesis, including vascular endothelial growth factor (VEGF) and platelet derived growth factor (PDGF). This study evaluated the potential wound healing activity of *Greyia radlkoferi* Szyszyl (GR) and its effect on growth factors regulating angiogenesis. The ethanolic leaf extract of GR was evaluated for antibacterial activity against wound associated bacteria; *Staphylococcus aureus* and *Pseudomonas aeruginosa*. It exhibited antibacterial activity against two strains of *S. aureus* (ATCC 25293 and ATCC 6538) displaying a minimum inhibitory concentration (MIC) at 250 and 500 μg/ml, respectively. The antioxidant activity of the extract was investigated for nitric oxide (NO) scavenging activity and showed a fifty percent inhibitory concentration (IC_50_) of 1266.5 ± 243.95 μg/ml. The extract was further investigated to determine its effect on the proliferation and modulation of growth factors secreted by human keratinocytes (HaCaT). Its effect on wound closure was evaluated using the scratch assay, where non-toxic concentrations were tested, as determined by the antiproliferative assay against HaCat cells (IC_50_ > 400 μg/ml). Results showed that the extract significantly inhibited wound closure, with a percentage closure of 60.15 ± 1.41% (*p* < 0.05) and 49.52 ± 1.43% (*p* < 0.01) at a concentration of 50 and 100 μg/ml, respectively, when compared to the 0.25% Dimethyl sulfoxide vehicle control (65.86 ± 1.12%). Quantification of secreted growth factors from cell-free supernatant, collected from the scratch assay, revealed that the extract significantly decreased the concentration of platelet-derived growth factor (PDGF-AA) at both 50 (*p* < 0.05) and 100 μg/ml (*p* < 0.001) (443.08 ± 77.36 and 178.98 ± 36.60 pg/ml) when compared to the 0.25% DMSO vehicle control (538.33 ± 12.64 pg/ml). Therefore, whilst the extract showed antibacterial activity against wound associated bacteria, it did not induce wound healing but rather showed a significant inhibition of wound closure, which was confirmed by the inhibition of PDGF-AA, a major growth factor involved in angiogenesis. Therefore, the GR extract, should be considered for further investigation of anti-angiogenic and anti-metastatic properties against cancer cells.

## 1 Introduction

Angiogenesis involves the proliferation and migration of endothelial cells which occurs concurrently with the formation of nascent epithelial tissue, as seen in the wound healing process. The formation of microvessels is vital for blood and nutrient supply to the nascent tissues to ensure healthy wound recovery and maturation of the epidermal layer. However, overstimulation of the angiogenic process may lead to the progression of pathological diseases, such as tumor growth and metastatic developments (Rajabi and Mousa, 2017). Cancerous cells secret an excess of pro-angiogenic factors, such as vascular endothelial growth factor (VEGF) and platelet-derived growth factor (PDGF-AA), which over-stimulate angiogenesis within the tumour, leading to exponential tumour growth and metastasis due to the continuous supply of blood and nutrients into the cancer mass (Lugano et al. 2020). The perpetual secretion of VEGF and PDGF-AA leads to a bias towards the formation of nascent blood capillaries as opposed to maturation of these capillaries, which results in a high permeability index of the surrounding tissue (Azzi et al., 2013). This provides metastatic cancer cells a mode of dissemination into the bloodstream which may lead to the formation of distal tumour sites (Carmeliet and Jain 2011).

Wound healing is an intricate process which involves various factors and physiological mechanisms, such as cell proliferation and angiogenesis (Aldridge 2015). Therefore, the rate of dermal wound healing is influenced by the proliferation of human keratinocytes and angiogenesis, which are both regulated by specific growth factors secreted by numerous somatic cells, including keratinocytes, throughout the wound healing process (Lynch et al. 1989) (Table 1).

**TABLE 1:** Human growth factors secreted by keratinocytes and their role in wound healing/angiogenesis.

Growth factor	Role of growth factor in wound healing/angiogenesis	Cells which produce growth factor	References
Angiopoietin-2 (ANGPT2)	Stimulates angiogenesis by preventing maturation of nascent microvessels	Endothelial cellsKeratinocytes	(Krikun et al. (2000))
Fibroblast growth factor (FGF)	Stimulates cell migration and angiogenesis by facilitating the secretion of degradative enzymes by endothelial cells	Epithelial/mesenchymal cells Keratinocytes	Barrientos et al. (2008)
Hepatocyte growth factor (HGF)	Stimulates mitosis of epithelial cells Facilitates angiogenesis by stimulating the secretion of proangiogenic factors	Fibroblasts Keratinocytes Mesenchymal cells Platelets	Li et al. (2013)
Macrophage colony-stimulating factor (M-CSF)	Regulates macrophage cell function and differentiation Stimulates the secretion of pro-angiogenic factors in endothelial cells	Bone marrow cells Endothelial cells Fibroblasts Keratinocytes Monocytes	Barrientos et al. (2008)
Platelet-derived growth factor (PDGF-AA)	Regulates endothelial cell proliferation and migration Stimulates production of collagen and other connective tissues which facilitate wound contraction Stimulates the production of pro-angiogenic factors by endothelial cells	Endothelial cells Keratinocytes Macrophage Platelets Smooth muscle cells	Lynch et al. (1989)
Vascular Endothelial Growth Factor (VEGF)	Stimulates proliferation of endothelial cells Stimulates angiogenesis by promoting epithelial cell proliferation and microvessel permeability	Fibroblasts Keratinocytes Macrophages Mesenchymal cells	Barrientos et al. (2008)
Erythropoietin (EPO)	Stimulates the proliferation and migration of epithelial cells Stimulates the production of pro-angiogenic factors	Fibroblasts Keratinocytes	(Haroon et al. (2003)

The rate of wound healing may be hindered due to complications such as wound infection, which disrupts healthy tissue regeneration and efficient angiogenesis due to severe swelling (Brem and Tomic-canic 2007). Wounds infected by bacteria such as *Staphylococcus aureus* and *Pseudomonas aeruginosa*, result in severe inflammation around the wound site (Bessa et al., 2013; Micek et al., 2005; Cohen and Kurzrock, 2004). Excessive swelling in nascent tissues may result in tissue tearing and restriction of blood flow which ultimately impairs the angiogenic and wound healing pathways (Brem and Tomic-canic, 2007). This highlights the importance of developing effective antibacterial agents that may facilitate healthy wound healing *via* the stimulation of growth factors associated with angiogenesis and endothelial cell proliferation and migration.

The potential of medicinal plants as a resource for novel therapeutic agents is made evident due to the use of these plants in various traditional medicinal practices across the globe (Ekor 2014). Several South African medicinal plants, such as various Aloe and Buchu species (*Agathosma* spp.), have been used in wound healing practices for generations around the world (Pereira and Bártolo 2016). The gel of aloe species, such as *Aloe arborescens* Miller and *Aloe ferox* Miller, is directly applied to dermal wounds and burns to provide soothing effects and promote wound healing (Jia et al., 2008; Morgan and Nigam, 2013). Tinctures of various *Agathosma* spp*.* are directly applied to dermal wounds and abrasions to provide antibacterial and anti-inflammatory effects (Van Wyk, 2011). This highlights the potential for drug discovery through investigation of unexplored plant species.

*Greyia radlkoferi* Szyszyl, belonging to the Melianthaceae family, is indigenous to various parts of southern Africa, with distributions across Limpopo, Mpumalanga and Kwa-Zulu Natal. This deciduous shrub can grow up to 5 m in height and sprouts red bottlebrush-like flowers during the winter and early spring seasons (Malele et al., 2021). Although commonly used in traditional practices to make utensils and textiles, the medicinal value of *G. radlkoferi* has not been fully investigated (De La Cruz, 2004). However, studies have revealed that extracts prepared from the leaves are rich in bioactive flavonoids, which have displayed significant activity against skin hyperpigmentation (Bohm and Chan, 1992; De Canha et al., 2013). Flavonoids have been identified for their potential to stimulate wound healing through various mechanisms, such as by modulation of the inflammatory response, angiogenesis, cell migration and oxidative stress (Carvalho et al., 2021). Although rich in flavonoid content, the effects of *G. radlkoferi* leaf extracts on wound healing have not been documented.

Therefore, the aim of this study was to investigate the antibacterial activity against *S. aureus* and *P. aeruginosa* and the wound healing potential of *G. radlkoferi* ethanolic (EtOH) leaf extract (GR-EtOH), and its effects on growth factors involved in wound healing and angiogenesis.

## 2 Methods and Materials

### 2.1 Materials and Reagents

PrestoBlue® was purchased from Life Technologies (Johannesburg, South Africa). The University of Cape Town (South Africa) donated the spontaneously immortalized human keratinocyte (HaCat) cell line. Strains of *S. aureus* (ATCC 6538 and ATCC 25293) and *P. aeruginosa* (ATCC 9027) were obtained from Anatech Analytical Technology (Johannesburg, South Africa). Cell culture reagents, including Dulbecco’s Modified Eagle Medium (DMEM), trypsin-EDTA (0.25%), fetal bovine serum (FBS), phosphate buffer saline (PBS), and antimicrobials, such as penicillin, streptomycin and amphotericin B, were supplied by ThermoFisher Scientific (Johannesburg, South Africa). Sterile cell culture flasks and multi-well plates were purchased from Lasec South Africa (Pty) Ltd. (Midrand, South Africa). Sodium nitroprusside, Nutrient Agar/Broth, Tryptic Soy Agar/Broth, vancomycin (purity ≥90%), ciprofloxacin (purity ≥98%), actinomycin D (purity ≥95%), ascorbic acid (purity >98%), Griess-Ilosvay’s nitrite reagent, HPLC-grade methanol, galangin (purity ≥99%) and all other chemicals and reagents were acquired from Sigma Chemicals Co. (St. Louis, MO, United States ). The LEGENDPlex™ Growth Factor Panel Flow cytometry Kit was supplied by Biocom Biotech (Pty) Ltd. (Centurion, South Africa).

### 2.2 Plant Material Collection and Extraction

Leaves of *G. radlkoferi* were collected in February 2018 from the Manie van der Schijff Botanical Garden (-25.752406, 28.229718) at the University of Pretoria. A herbarium specimen number (PRU 122434) was prepared and submitted at the H.G.W.J. Schweickerdt Herbarium, University of Pretoria, South Africa. The fresh leaf material (205 g) was blended with 2 L of absolute ethanol until homogenized. Thereafter, the mixture was filtered using a Buchner funnel (No three Whatman filter paper) and the filtrate was evaporated with a Bϋchi Rotary evaporator. The resulting semi-liquid extract was then freeze-dried using a freeze dryer (ICHR101530, Alpha one to two Ldplus (Lasec SA (Pty) Ltd.), until a powdered extract was obtained (6.22% yield).

### 2.3 High-Performance Liquid Chromatography (HPLC) of GR-EtOH Extract

**FIGURE 1:**
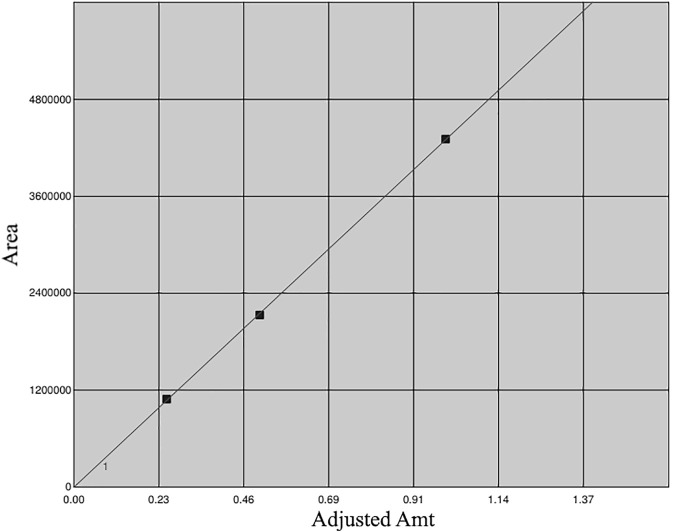
Calibration curve of galangin generated using 0.25, 0.50 and 1.00 mg/ml.

Previous reports have indicated the presence of galangin in an ethanolic leaf extract of *G. radlkoferi* (Lall et al., 2016). However, quantification of galangin from the ethanolic leaf extract has not yet been reported. Therefore, a gradient HPLC method was used to quantify galangin in GR-EtOH using a prepacked reverse-phase Luna Synergi 4u Fusion-80A column (150 × 4.6 mm, particle size 4 μm) (Separations, Randburg, South Africa) equipped with a UV detector (270 nm) and operating at 25°C. Samples, dissolved in methanol, were filtered through a 0.2 μm syringe filter prior to injection. An injection volume of 20 μL per sample was used with a constant flow rate of 1.2 ml/min at 25°C. Data acquisition was performed over a 2 h period. The mobile phase gradient (Table 2) consisted of water +0.5% acetic acid (solvent A) and acetonitrile +0.5% acetic acid (solvent B). The percentage galangin quantified in GR-EtOH was calculated from a galangin standard curve (Figure 1).

**TABLE 2:** | Gradient of the mobile phase used for elution of galangin in the ethanolic leaf extract of *Greyia radlkoferi*.

Time (min)	Solvent A (%)	Solvent B (%)
0	80	20
2.5	80	20
30	20	80
120	80	20

### 2.4 Antibacterial Assay

Pure cultures of *S. aureus* (ATCC 6538 and ATCC 25293) and *P. aeruginosa* (ATCC 9027) were maintained on sterile tryptic soy and nutrient agar plates, respectively. The antibacterial activity of GR-EtOH was investigated following the method described by Lall et al. (2013). Subcultures of *S. aureus* and *P. aeruginosa* were inoculated at 1.5×10^8^ colony forming units per mL (CFU/ml) in correlation to the 0.5 McFarland standard, and subsequent inoculations were further diluted (1:1000). A stock concentration of GR-EtOH was prepared at 2 mg/ml (in 10% DMSO) and was further serially diluted in the respective broth at final concentrations ranging from 7.81–500 μg/ml (with resulting DMSO concentrations of 0.03–1%, respectively). The positive controls, vancomycin and ciprofloxacin were tested at final concentrations ranging from 0.39–50 μg/ml and 0.039–5 μg/ml against *S. aureus* and *P. aeruginosa*, respectively. Media without bacteria, a DMSO vehicle control (ranging from 0.03–1%) and an untreated bacterial control were included. After the addition of bacteria, the plates were incubated for 18 h at 37°C, thereafter, 20 µL of PrestoBlue® viability reagent was added to the wells and the minimum inhibitory concentration (MIC) was determined by visual examination, after 20 min of incubation.

### 2.5 Cell Culture

#### 2.5.1 Culture Growth and Maintenance

The HaCaT cells were maintained in DMEM supplemented with 10% heat-inactivated fetal bovine serum, 1% antibiotics (100 U/mL penicillin and 100 μg/ml streptomycin), and 1% amphotericin B (250 μg/ml) at 37°C and 5% CO_2_. The cells were sub-cultured after treatment with trypsin-EDTA (0.25% trypsin containing 0.01% EDTA) for 5 min, after an 80% confluent monolayer had formed.

#### 2.5.2 Antiproliferative Assay

The antiproliferative assay was performed according to the method described by Lall et al. (2013). The HaCaT cells were seeded at a concentration of 1×10^5^ cells/mL (10,000 cells/well) in sterile 96-well micro-titre plates (with 100 µL of cell suspension per well). The plates were incubated for 24 h to allow cell attachment to occur. Media controls, with and without cells were used as 100 and 0% cell viability controls, respectively. Dimethyl sulfoxide (DMSO) was included as a toxic inducer at final concentrations ranging from 0.625 to 20% and as a vehicle control (1%). A stock concentration of GR-EtOH was prepared at a concentration of 40 mg/ml (in DMSO). Thereafter, the extract was serially diluted in complete media, and tested at final concentration ranging from 3.13–400 μg/ml. All wells were tested at a final volume of 200 µL per well. The plates were incubated for a further 72 h at 37ºC and 5% CO_2_. PrestoBlue® viability reagent was then added (20 µL) and the plates were incubated for 2 h to allow for a colour change to occur. The fluorescence of the resulting colour complex was measured (excitation of 560 nm and emission of 590 nm) using a Victor® Nivo™ Multimode Microplate Reader (PerkinElmer South Africa (Pty) Ltd.). The percentage cell viability was determined using the formula:

$$\text{\% Cell viability}=\left[\frac{\left({\text{A}}_{\text{treatment}}\;-{\text{ A}}_{\text{blank}}\right)}{\left({\text{A}}_{\text{control}}\;-{\text{ A}}_{\text{blank}}\right)}\right]\times 100\;\%$$

Where, A = Fluorescence, A_blank_ = fluorescence of media without cells (0% control), A_control_ = fluorescence of DMSO (1%) vehicle control and A_treatment_ = fluorescence of sample. The 50% inhibitory concentration (IC_50_) of GR-EtOH and the DMSO toxic inducer control was determined using the GraphPad Prism Version 4.0 software (San Diego, California, United States ).

### 2.6 Nitric Oxide Scavenging Assay

The nitric oxide (NO) scavenging assay was performed according to the method described by Mayur et al. (2010). The GR-EtOH extract and positive control (ascorbic acid) were prepared at stock concentration of 8000 μg/ml and 2000 μg/ml (in DMSO), serially diluted in ethanol and tested at final concentrations ranging from 31.25–4000 μg/ml and 15.63–2000 μg/ml, respectively. DMSO was used as the vehicle control. A 10 mM solution of sodium nitroprusside (50 µL) was added to all wells and the plates were subsequently incubated for 90 min at room temperature. Thereafter, 100 μL Griess reagent was added to all the wells. Blank colour controls were prepared for the GR-EtOH and the positive control, where 100 µL of distilled water was added to the wells instead of Griess reagent. After incubation for 5 min, the absorbance was measured at 546 nm using a BIO-TEK Power-Wave XS multi-well reader (A.D.P, Weltevreden Park, South Africa) using the KC Junior software. The percentage NO inhibition (%) was calculated using the following equation:

$$\text{\% NO inhibition}=\left[\frac{{\text{A}}_{\text{vehicle}-}\left({\text{A}}_{\text{t}}\;-{\text{ A}}_{\text{tb}}\right)}{{\text{A}}_{\text{vehicle}}}\right]\times 100\;\%$$

Where A_vehicle_ = average DMSO vehicle control absorbance, A_t_ = absorbance of the sample and A_tb_ = absorbance of the sample blank colour control. The concentration required to scavenge 50% of produced NO (IC_50_) was determined thereafter using GraphPad Prism Version 4.0 software.

### 2.7 Scratch Assay and Growth Factor Quantification

The effect of GR-EtOH on the proliferation of HaCat cells was investigated using the scratch assay according to a method described by Liang et al. (2007), with slight modifications. The HaCat cells were seeded at a concentration of 1.5×10^5^ cells/mL (150,000 cells/well) in 24-well micro-titre plates and incubated for 24 h at 37ºC and 5% CO_2_ to form a confluent monolayer. A 1000 μL pipette tip was used to scratch a cross in the monolayer to simulate a wound and the media was subsequently replaced to remove cell debris. The extract was tested at two non-toxic concentrations (50 and 100 μg/ml), as determined from the antiproliferative assay. Media controls, with and without cells, and a DMSO (0.25%) vehicle control were included in the experiment. Immediately after addition of GR-EtOH and the controls, images were taken of the scratches at 0 h incubation. The plates were subsequently incubated for 18 h, after which images were taken again. Images were analysed using ImageJ Version 1.53e software to calculate the percentage wound closure after 18 h incubation. The analysis protocol for each image at 0 and 18 h incubation occurred as follows; the image type was set to 8-bit and a bandpass filter was applied. The threshold was adjusted to the automatic setting and a radius filter was applied with a minimum radius of seven or above, until the outline of the scratch was prominent. Thereafter, the wand tool was used to select the scratch border and the analyse-measure function was used to obtain the area of the selected scratch border, and the results were recorded. Percentage wound closure was calculated using the following equation:

$$\text{\% Wound closure}=\left[\frac{\left({\text{A}}_{0\text{h}}\;-{\text{ A}}_{18\text{hr}}\right)}{{\text{A}}_{0\text{h}}}\right]\times 100\;\%$$

(where, A = area of the scratch)

After images were taken at 18 h incubation, the plates were centrifuged (980 rpm) and supernatant was collected from each of the wells and frozen at -80ºC until use for quantification of growth factors, which was performed using the LEGENDPlex™ Human Growth Factor Panel Kit from BioLegend. PrestoBlue® was added to the remaining media and measured as described in the antiproliferative assay to calculate the percentage cell viability to ensure that GR-EtOH and the controls did not show toxicity (Lall et al., 2013). Quantification of growth factors from the cell free supernatant was conducted according to the manufacturer’s protocols (Cat # 741061) Briefly, 25 µL supernatant and standards (tested at concentration ranging from 0–10,000 pg/ml (M-CSF) and 0–50,000 pg/ml (ANGPT2, EPO, FGF, HGF, PDGF-AA and VEGF) were added to the V-bottom 96-well micro-titre plate, followed by the additional of 25 μL assay buffer, 25 µL of pre-mixed beads and 25 μL detection antibodies to all the wells. The plate was incubated at room temperature for 2 h with constant shaking at 800 rpm. After incubation, 25 μL streptavidin-phycoerythrin (SA-PE) conjugate was added to each well followed by 30 min incubation with constant shaking at 800 rpm. The plate was then centrifuged at 4000 rpm for 5 min and the supernatant removed (without disruption of the bead pellet). The plate was washed by adding 200 µL of wash buffer to each well where after the plate was centrifuged and the supernatant was removed. The beads were resuspended in 150 μL wash buffer and the wells were read on a BD Accuri™ C6 Plus Flow Cytometer BD Biosciences (San Diego, CA, United States ) according to the LEGENDPlex™ protocol (Biolegend, 2021). The number of beads acquired was set to 2000–2500 on Beads A gate and B gate, and the flow rate was set to slow. The obtained data was analysed using the LEGENDPlex™ v8.0 data analysis software to determine the concentration of growth factors (pg/ml).

### 2.8 Statistical Analysis

The antibacterial assay was performed in duplicate with three independent experiments (*n* = 2), whereas the antiproliferative and NO scavenging assays were performed in triplicate with three independent experiments (*n* = 3). The scratch assay was performed in duplicate with two separate experiments (*n* = 2) and the quantification of growth factors was performed in triplicate (*n* = 3). The results were displayed as mean ± standard deviation. The IC_50_ from the NO and antiproliferative assay were calculated using non-linear regression analysis of the sigmoidal dose-response curves with constraints set at 100 (top) and 0 (bottom) using GraphPad Prism Version 4.0 software, whereas the MIC values were visually interpreted. Significance of results was determined using one-way ANOVA when compared to the 0.25% DMSO vehicle control (+), followed by Dunnett’s multiple comparison test or Bonferroni posttest (as indicated in the results section) using GraphPad Prism Version 4.0 software, where *p < 0.05 and **p < 0.01 indicates statistical significance.

## 3 Results

### 3.1 HPLC Quantification of Galangin

**FIGURE 2:**
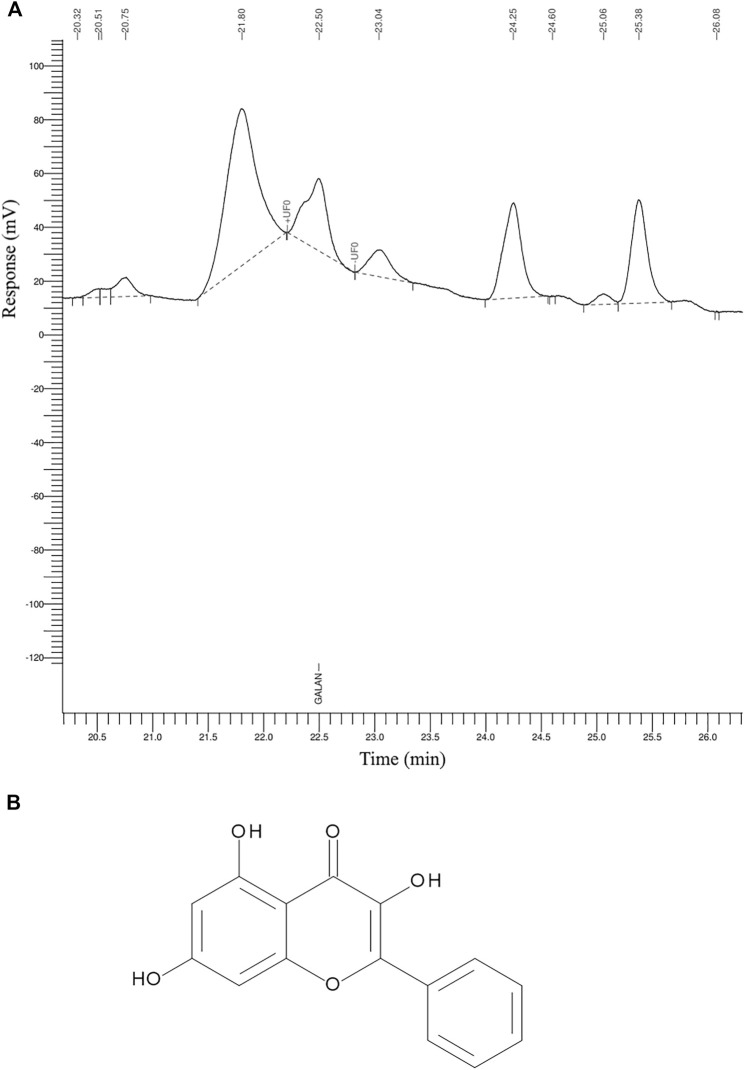
The HPLC chromatogram of **(A)** *G. radlkoferi* EtOH leaf extract used for the quantification of **(B)** galangin.

The GR-EtOH was subjected to analytical separation by HPLC for the quantification of galangin. The results exhibited that GR-EtOH yielded a 2.15% composition of galagin, a known compound isolated from the leaves of *G. radlkoferi* (Figures 2A, B) (Lall et al., 2016). The representative galangin peak eluted at time 22.5 min (Figure 2A).

### 3.2 Antibacterial Activity

PrestoBlue® is a viability reagent which changes in colour from blue to pink in the presence of viable cells, due to the reduction of resazurin to resorufin by viable cellular oxidoreductase enzymes (Lall et al., 2013). Visual MIC interpretation showed that GR-EtOH exhibited antibacterial activity against *S. aureus* ATCC 25293 (MIC of 250 μg/ml) and ATCC 6538 (MIC of 500 μg/ml), however did not display antibacterial activity against *P. aeruginosa* (ATCC 9027) at the highest tested concentration (MIC >500 μg/ml). The vancomycin positive control exhibited an MIC of 0.78 μg/ml against both strains of *S. aureus* and the ciprofloxacin positive control exhibited an MIC of 0.08 μg/ml against *P. aeruginosa*.

### 3.3 Antiproliferative Activity

**FIGURE 3:**
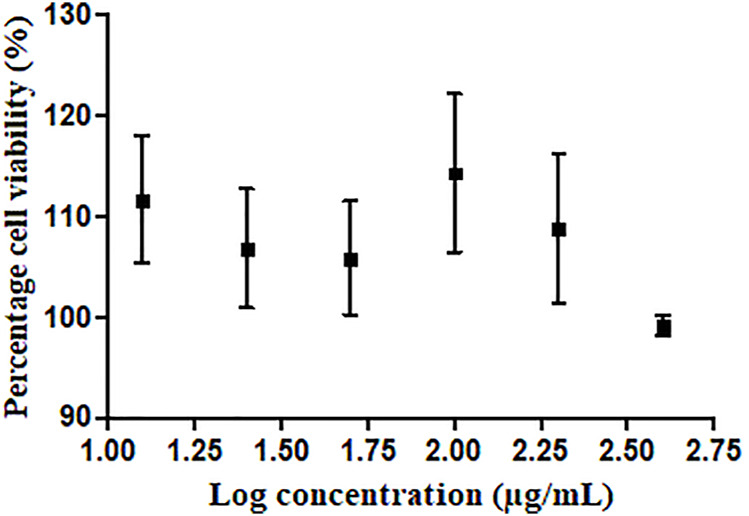
Dose-response curve depicting HaCat cells treated with *G. radlkoferi* EtOH leaf extract.

The antiproliferative assay was performed to determine the potential toxicity of GR-EtOH against normal human keratinocytes (Figure 3). In addition, results from the assay were used to identify non-toxic concentrations at which to conduct the scratch assay and growth factor quantification. The results indicated that GR-EtOH did not display cytotoxic effects on HaCat cells at the highest tested concentration (IC_50_ > 400 μg/ml), indicating that the extract had low (300 μg/ml < IC_50_ < 1000 μg/ml) to no (IC_50_ > 1000 μg/ml) toxicity (Kuete and Efferth, 2015). The DMSO toxic inducer exhibited an IC_50_ of 4.08 ± 1.13%.

### 3.4 Nitric Oxide Scavenging Activity

The NO scavenging activity for GR-EtOH was investigated to evaluate the potential antioxidant activity within the wound healing process. The positive control, ascorbic acid, exhibited an IC_50_ value of 60.32 ± 7.83 μg/ml, whereas the GR-EtOH exhibited an IC_50_ of 1266.5 ± 243.95 μg/ml.

Scratch assay to determine the effect on wound closure.

**FIGURE 4:**
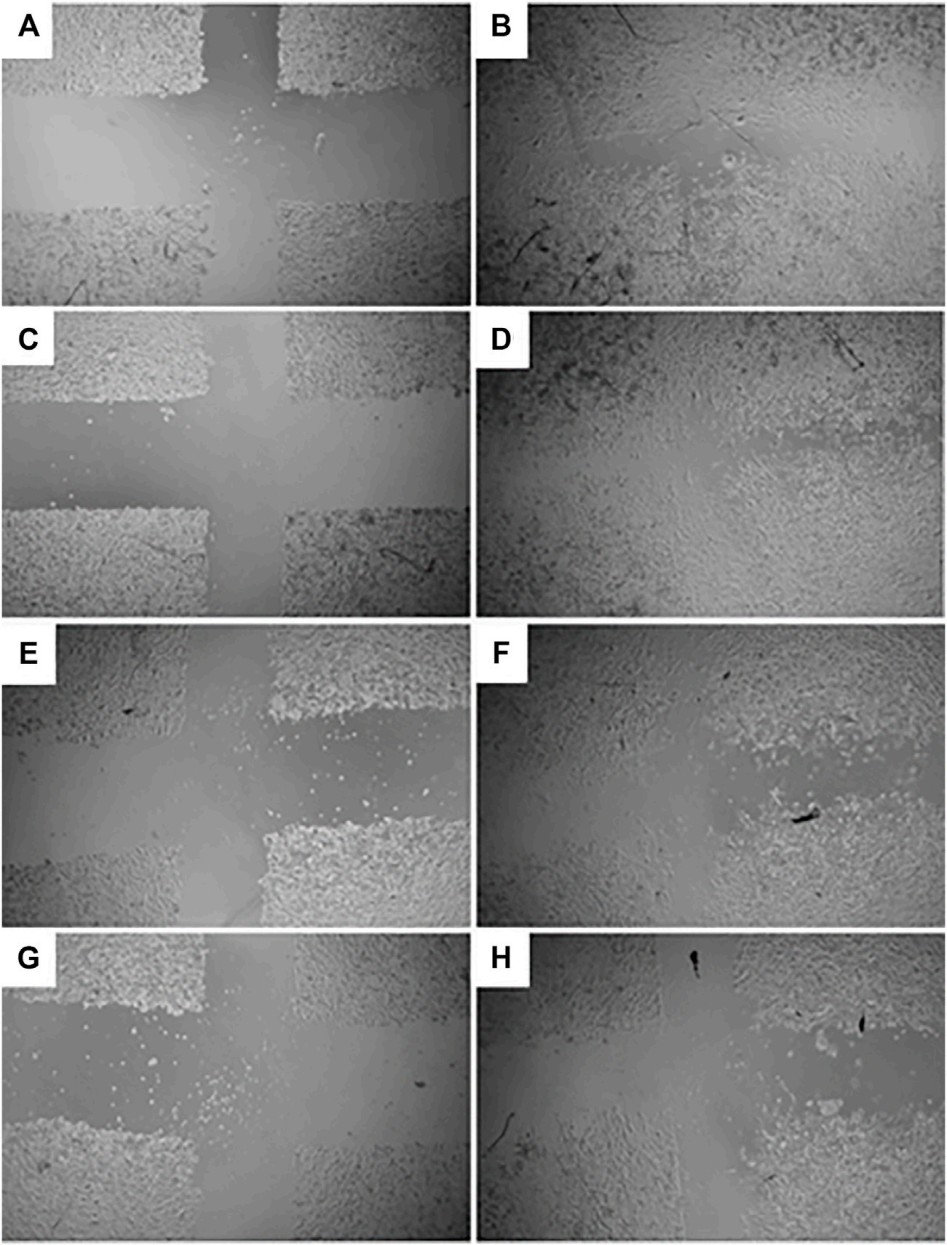
Images (×4 magnification) representing wound closure at 0 and 18 h incubation time of **(A)** untreated media control at 0 h and **(B)** after 18 h (C) 0.25% DMSO vehicle control after 0 h and **(D)** after 18 h **(E)** *G. radlkoferi* EtOH leaf extract (50 μg/ml) after 0 h and **(F)** after 18 h incubation **(G)** *G. radlkoferi* EtOH leaf extract (100 μg/ml) after 0 h and **(H)** after 18 h incubation.

The GR-EtOH extract was evaluated for potential wound healing activity using HaCat cells, which provides insight into the effect on cellular proliferation and migration. The extract was tested at two non-toxic concentrations (50 and 100 μg/ml), as determined by the antiproliferative assay. Images were taken at 0 and 18 h after incubation to determine the percentage wound closure (Figure 4). GR-EtOH (at 50 and 100 μg/ml) showed significant inhibition of wound closure with a percentage closure of 60.15 ± 1.41% (*p <* 0.05) and 49.52 ± 1.43% (*p* < 0.01), respectively when compared to the 0.25% DMSO vehicle control, which showed a percentage wound closure of 65.86 ± 1.12%. In addition, the 0.25% vehicle control did not show significant difference in wound closure when compared to the media (untreated) control (65.38 ± 1.61%) (Figure 4).

### 3.5 Growth Factor Quantification

**FIGURE 5:**
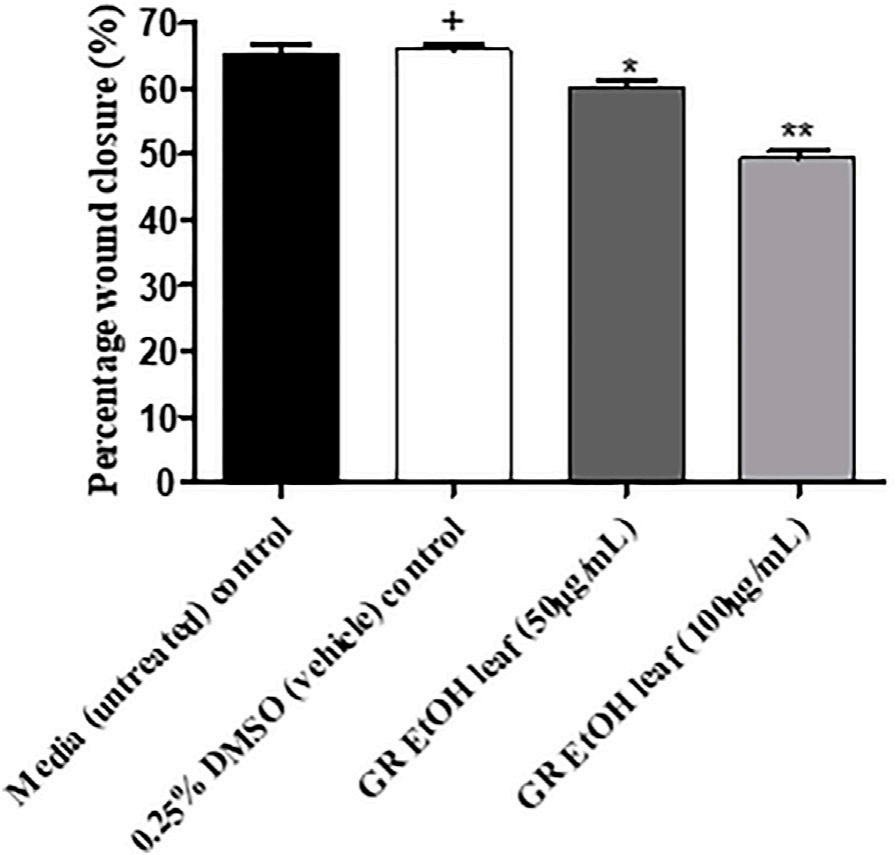
Percentage wound closure (%) of *G. radlkoferi* EtOH leaf extract (50 and 100 μg/ml) on HaCat cells after 18 h incubation. Values are displayed as mean ± standard deviation (*n* = 2), where *p < 0.05 and **p < 0.01 indicated statistical significance (one-way ANOVA and Dunnett’s multiple comparison test) when compared to the 0.25% DMSO (vehicle) control (+). No statistical difference was determined between the media (untreated) control and the 0.25% DMSO (vehicle) control.

**FIGURE 6:**
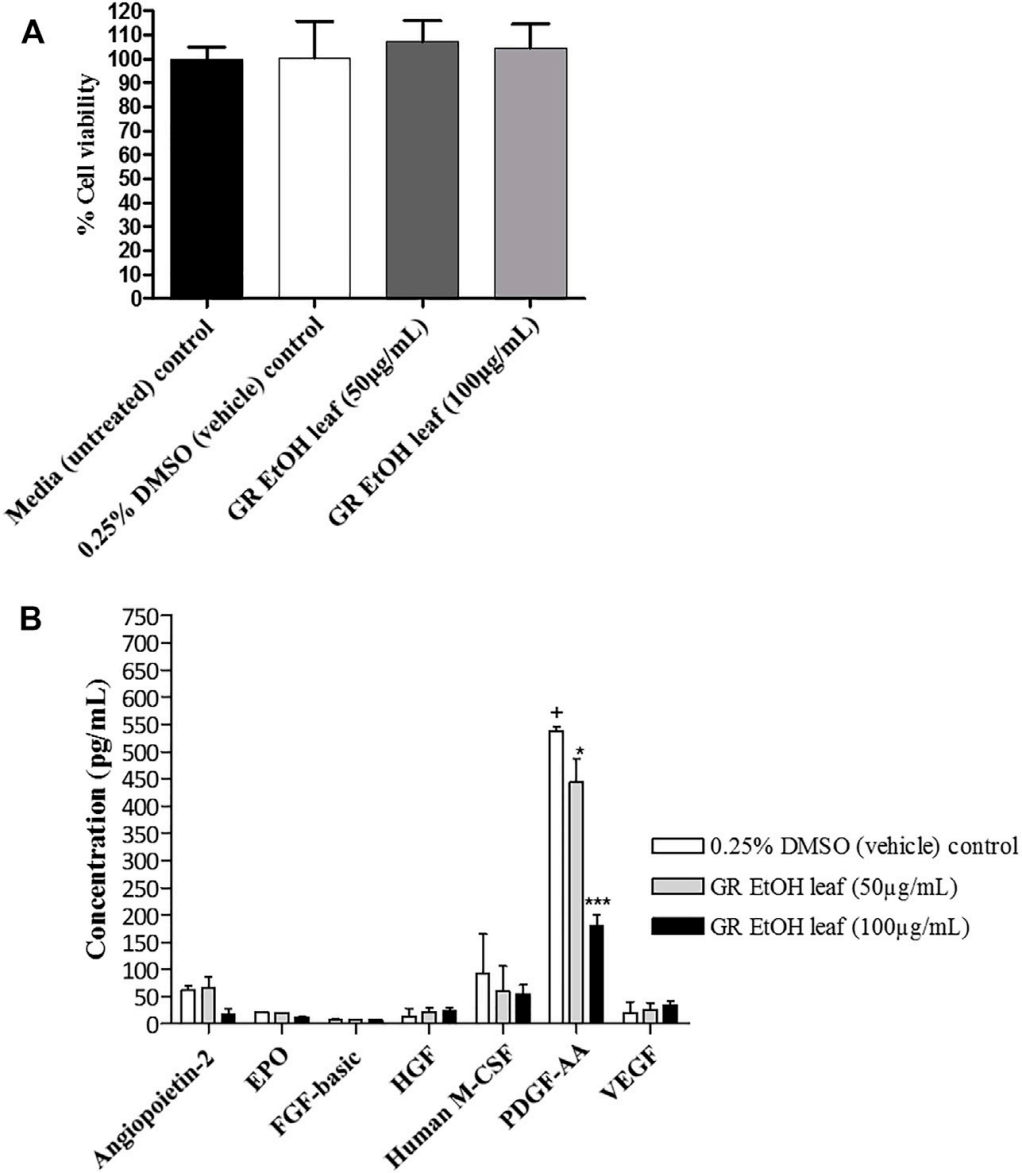
**(A)** Cell viability (%) of human keratinocytes (HaCat) treated with *G. radlkoferi* EtOH leaf extract (at 50 and 100 μg/ml) after 18 h. Controls included cells grown in media (untreated) and cells treated with 0.25% DMSO (vehicle) control. Data shown are mean ± SD (*n* = 2). No statistical significance was observed when compared to untreated cells. Statistical analysis was done using one-way ANOVA followed by Dunnett’s multiple comparison test **(B)** The effect of *G. radlkoferi* EtOH leaf extract (at 50 and 100 μg/ml) on the production of human growth factors (pg/ml) in HaCat cells after 18 h incubation. Data shown are mean ± SD (*n* = 3). Statistical analysis was done using one-way ANOVA followed by Bonferroni’s posttest, where *p < 0.05 and ***p < 0.001 indicated statistical significance when compared to the 0.25% DMSO (vehicle) control (+).

The quantification of growth factors secreted by HaCat cells (cell-free supernatant collected from the scratch assay) after exposure to GR-EtOH (50 and 100 μg/ml) was evaluated. Statistical analysis showed that the 0.25% DMSO (vehicle) control did not have an effect on growth factor production when compared to the media (untreated) control. Cell viability was not significantly affected by GR-EtOH at 50 and 100 μg/ml when compared to the 0.25% DMSO (vehicle) and media (untreated) controls, thereby establishing that the modulation of growth factors was due to the extract and not affected by cell viability (Figure 6A). The results revealed that GR-EtOH significantly decreased the concentration of PDGF-AA at both 50 (*p <* 0.05) and 100 μg/ml (*p <* 0.001) (443.08 ± 77.36 and 178.98 ± 36.60 pg/ml, respectively) when compared to the 0.25% DMSO vehicle control (538.33 ± 12.64 pg/ml) (Figure 5B). Furthermore, results showed that GR-EtOH decreased ANGPT2 (at 100 μg/ml), and M-CSF (at 50 and 100 μg/ml) production, however, this was not statistically significant (Figure 6B).

## 4 Discussion

The potential wound healing activity of GR-EtOH was evaluated by determining its antibacterial, antioxidant and wound healing activity as well as its effect on the production of growth factors associated with angiogenesis. The extract exhibited antibacterial activity against two strains of *S. aureus* (Gram-positive bacteria) but not *P. aeruginosa* (Gram-negative bacteria). Previous studies conducted on three *Greyia* species (*G. radlkoferi*, *G. flanagani* Bolus and *G. sutherlandii* Hook. and Harv.) showed that EtOH leaf extract of *G. radlkoferi* has a rich flavonoid content with similar quantities of kaempferol and quercetin 3-*0*-mono- and diglycosides (Bohm and Chan, 1992). Various reports state that the antibacterial mechanism of flavonoids may be attributed to damage of the phospholipid bilayers of the bacterial cell wall in Gram-positive bacteria, however, the presence of the lipopolysaccharide layer renders Gram-negative bacteria less susceptible to these compounds (Recio and Rios, 2005; Osonga et al., 2019; Yuan et al., 2021). There are numerous studies which indicate that kaempferol and quercetin glycosides display antibacterial activity against Gram-positive and Gram-negative bacteria, including strains of *S. aureus* and *P. aeruginosa*, through the inhibition of biofilm formation by reducing the expression of adhesion-related genes, and the inhibition of efflux pump activity in the bacterial cell wall (Falcão-Silva et al., 2008; Ming et al., 2017; Wang et al., 2018; Pal and Tripathi, 2020). Therefore, the antibacterial activity of GR-EtOH displayed against Gram-positive *S. aureus* may be attributed to a similar mechanism of action exhibited by the previously mentioned compounds.

The GR-EtOH extract showed low to no toxicity (IC_50_ > 400 μg/ml) against HaCat cells in correspondence with thresholds set by Kuete and Efferth (2015) (Figure 2). Using the scratch assay, to determine the potential wound healing activity, GR-EtOH showed a significant decrease in wound closure (Figures 4, 5). At 100 μg/ml, the extract exhibited a lower percentage wound closure than at 50 μg/ml. There are several reports which indicate that kaempferol and quercetin both display conflicting evidence in their potential effects on wound healing; kaempferol stimulates the migration of keratinocytes through the activation of the focal adhesion kinase (FAK) and Ras-related C3 botulinum toxin substrate 1 (Rac1) cell signaling pathways, whereas quercetin inhibits keratinocyte migration by inhibiting transforming growth factor β1 (TGF-β1)-induced epithelial-mesenchymal transition (EMT) (Petpiroon et al., 2015; Kim et al., 2020). In addition, the extract exhibited low NO scavenging potential with an IC_50_ of 1266.5 ± 243.95 μg/mL. A study by Lall and Sharma (2014), showed that an ethanolic leaf extract of *G. sutherlandii* displayed low toxicity towards B16-F10 mouse melanoma cells (IC_50_ of 107.85 ± 1.53 μg/ml) in comparison to the positive control, actinomycin D (IC_50_ of 4.5 × 10^−3^ ± 0.5 × 10^−3^ μg/ml). The study further showed that the extract displayed DPPH radical scavenging activity with an IC_50_ of 7.9 ± 0.23 μg/ml. Another study conducted by Mapunya et al. (2011) indicated that the ethanolic leaf extract of *G. flanaganii* inhibited melanin production through the inhibition of the tyrosinase enzyme (IC_50_ of 32.62 μg/ml), with low toxicity against B16-F10 melanocytes (IC_50_ > 400 μg/ml). These results may indicate that *Greyia* species potentially share a common mechanism of action in the inhibition of cell migration against melanocytes while exhibiting low toxicity. Furthermore, the species possess NO and DPPH radical scavenging activity which may indicate a potential inhibitory effect on angiogenesis by inhibition of blood vessel permeabilization and promotion of blood vessel maturation (hypothesised to be due to pericyte apoptosis caused by oxidative stress) (Radomska-Lesniewska et al., 2017). Therefore, this suggests potential anti-metastatic properties of different *Greyia* species which have yet to be explored. Further investigations of potential antioxidant mechanistic studies of *G. radlkoferi* should focus on the intracellular inhibition of NO synthesis by inhibition of macrophage nitric oxide synthase (iNOS) in murine macrophages (RAW 264.7) (Esposito et al., 2014). High levels of iNOS have been associated with tumour aggressiveness and decreased survival rates in patients diagnosed with triple negative breast cancer (Granados-Principal et al., 2015). Therefore, evaluating the effect of *G. radlkoferi* extract to inhibit iNOS may provide possible insight to the anti-cancer potential of the extract in breast cancer.

In order to identify the potential mechanism of action associated with inhibition of wound closure, cell-free supernatants from the scratch assay were further subjected to growth factor quantification to determine the effect of the extract on production of growth factors by keratinocytes associated with wound healing and angiogenesis. The GR-EtOH extract (at 50 and 100 μg/ml) displayed significant inhibition in the production of the PDGF-AA growth factor (Figure 5B). Human PDGF-AA is a potent mitogen that plays a role in regulation of cell growth and proliferation in numerous cells, including human keratinocytes (Pietras et al., 2003). Therefore, the inhibition of PDGF-AA production by GR-EtOH correlates with the significant decrease in percentage wound closure (Figure 5; Figure 6B). This corresponds to higher inhibition of wound closure within the scratch assay where treatment with 100 μg/ml resulted in a lower increase in percentage wound closure than treatment with 50 μg/ml. Human PDGF-AA, along with epithelial cell proliferation and migration, also plays a significant role in the stimulation of angiogenesis. Thus there is increasing evidence of a promising target of this cellular factor in cancer treatment through inhibition of tumor angiogenesis (Pietras et al., 2003). The results displayed that GR-EtOH inhibited PDGF-AA production, suggesting the potential *in vitro* anti-angiogenic mechanism of this extract. Isolation studies conducted on the ethanolic (EtOH) leaf extract of *G. radlkoferi* revealed the following compounds were present in the extract; chalcone, galangin, genistein, pinocembrin, and 2′,6′-dihydroxy-4′-methoxydihydrochalcone (Lall et al., 2016). Chalcone, and derivatives of the compound, have demonstrated anti-angiogenic potential by inhibition of expression of VEGF and matrix metalloproteinases (MMPs), and have displayed inhibition of various cell signaling pathways which play a role in angiogenesis, such as the NFκB, PI3-K/Akt, and ERK1/2 signaling pathways (Mojzis et al., 2008). Although the mechanism of action is not fully understood, galangin has been shown to inhibit endothelial cell migration and angiogenesis by downregulating CD44, a multifunctional cell surface glycoprotein which is over expressed in glioma (brain cancer) (Chen et al., 2019). Furthermore, the HPLC analysis of GR-EtOH exhibited elution of galangin at time 22.5 min, therefore confirming the presence of this compound within the extract (2.15%) which may contribute to the inhibitory activity of the extract in HaCaT cells. Galangin has been previously investigated in numerous studies and has exhibited anti-angiogenic and metastatic potential in liver cancer (HepG2) and ovarian cancer (OVCAR-3) cells, via suppression of the PKC/ERK and Akt/p70S6K/HIF-1α signaling pathways, respectively (Chien et al., 2015; Huang et al., 2015). These findings may substantiate to investigate this extract for anti-metastatic potential. Genistein has also displayed anti-angiogenic potential by inhibiting the expression of FGF in vasoendothelial (BBCE) cells, as well as inhibition of migration of these cells (Fotsis et al., 1993). There are no reports of bioassays conducted on pinocembrin in order to evaluate the effect of the compound on angiogenesis, however, computational studies indicate that the compound may display anti-angiogenic potential by inhibiting VEGF Receptor-2, a perpetuator of tumor angiogenesis (Sharma et al., 2018).

In conclusion, whilst the *G. radlkoferi* EtOH leaf extract displayed antibacterial activity against wound associated bacteria, it did not induce wound healing but rather showed a significant inhibition of wound closure, which correlated with the inhibition of PDGF-AA, a vital growth factor involved in angiogenesis. Furthermore, these studies may highlight the anti-angiogenic potential of the *G. radlkoferi* EtOH leaf extract which has yet to be evaluated. Therefore, further investigation on the *in vivo* effects of *G. radlkoferi* on blood vessel formation should be evaluated using the chorioallantoic membrane (CAM) assay (Aleksandrowicz and Herr, 2015). In addition, *G. radlkoferi* should be considered for further evaluation of anti-angiogenic and anti-metastatic properties using cancer cell lines with metastatic potential, such as melanoma and breast cancers.

## Data Availability

The raw data supporting the conclusion of this article will be made available by the authors, without undue reservation.

## Abbreviations

ANGPT2, Angiopoietin-2; ANOVA, Analysis of variance; CAM assay, Chick Chorioallantoic Membrane assay; CFU, Colony forming units; DMEM, Dulbecco’s Modified Eagle Medium; EPO, Erythropoietin; EtOH, ethanol; FBS, Foetal Bovine Serum; FGF, Fibroblast growth factor; HaCat, Spontaneously immortalized human keratinocytes; HGF, Hepatocyte growth factor; IC50, 50% inhibitory concentration; iNOS, Nitric oxide synthase; M-SCF, Macrophage colony-stimulating factor; MIC, Minimum inhibition concentration; NO, Nitric oxide; PBS, Phosphate buffer saline; PDGF-AA, Platelet-derived growth factor; RBCs, Red blood cells; SA-PE, StreptAvidin-PhycoErythrin; VEGF, Vascular Endothelial Growth Factor; WBCs, White blood cells; WHO, World Health Organization; ZOI, Zone of inhibition.
